# An Unusual Case of Laryngeal Paraganglioma in a Patient with Carotid Body Paraganglioma: Multimodality Imaging Findings

**DOI:** 10.1155/2015/342312

**Published:** 2015-11-15

**Authors:** Serap Dogan, Serkan Senol, Hakan Imamoglu, Ummuhan Abdulrezzak, Afra Ekinci, Imdat Yuce, Mustafa Ozturk

**Affiliations:** ^1^Department of Radiology, Erciyes University Medical Faculty, Kayseri, Turkey; ^2^Department of Nuclear Medicine, Erciyes University Medical Faculty, Kayseri, Turkey; ^3^Department of Otorhinolaryngology, Erciyes University Medical Faculty, Kayseri, Turkey

## Abstract

Multiple paragangliomas of the head and neck are rare conditions. Carotid paragangliomas are most common multiple paragangliomas. Laryngeal paragangliomas are very rare neuroendocrine tumors and usually are seen as symptomatic solitary lesions. We present multimodality imaging findings of incidentally detected laryngeal paraganglioma in a woman with synchronous carotid body paraganglioma and positive family history. To the best of our knowledge, this is the first case of laryngeal and carotid body paragangliomas in a patient with positive family history. Radiologists should keep in mind that paragangliomas may occur in various locations as multiple tumors.

## 1. Introduction

Head and neck paragangliomas (HNP) are rare neuroendocrine neoplasms. They represent 0.03% of all neoplasms and 0.6% of head and neck neoplasms [1]. The most common locations of HNP are carotid body, jugular bulb, tympanic plexus, and vagal ganglia. Other less common sites are nose, paranasal sinus, nasopharynx, larynx, parotid gland, orbit, thyroid, and thoracic inlet. Although paragangliomas are usually solitary, multiple tumors may occur in approximately 10% of sporadic tumors and 40% of the familial variety [2]. Carotid body with jugular and/or vagal paraganglioma, tympanic with vagal paraganglioma, has been reported as most common combinations [3, 4]. To the best of our knowledge there are only two cases of laryngeal with carotid body paraganglioma as a rare combination [5, 6]. Familial relationship was not mentioned in these cases. Herein we present a case of laryngeal with carotid body paraganglioma in a patient with positive family history.

## 2. Case Report

A 32-year-old woman presented with swelling in the left neck for the past 1.5 years. She was a nonsmoker and nondrinker. Her past medical history was negative but her mother was operated on for carotid body paraganglioma. On physical examination, mobile, soft, 1.5 cm mass was palpated anterior to the sternocleidomastoid muscle on left side. There were no cranial nerve palsies or neck lymphadenopathy. A thorough review of other systems was negative. Laryngoscopic examination was not remarkable. Blood and urine catecholamine levels were normal.

**Figure 1:**
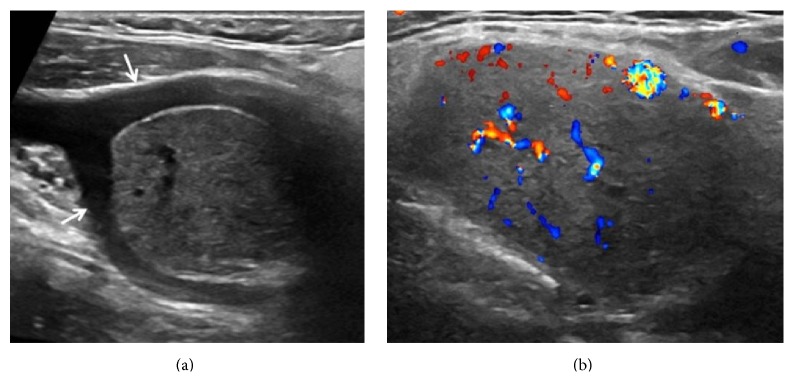
Gray-scale ultrasound (a) shows well defined, solid mass that causes splaying of the internal and external carotid arteries (arrows). High vascularity of lesions is seen in Doppler ultrasound image (b).

**Figure 2:**
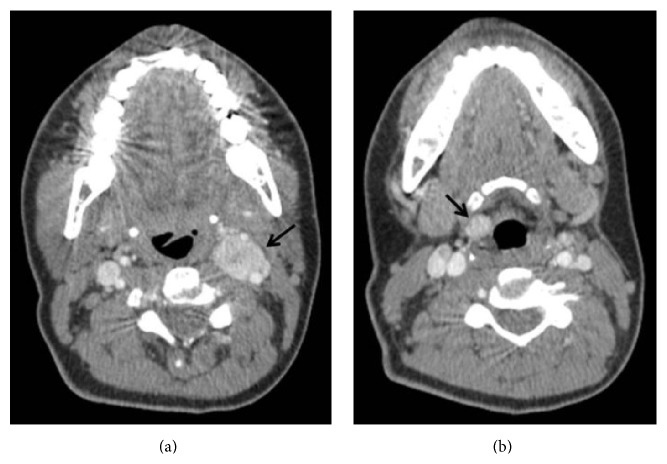
Axial contrast enhanced computed tomography demonstrates vascular mass (arrow) at left carotid bifurcation (a). Right supraglottic, well defined laryngeal mass (arrow) is seen in lower level contrast enhanced computed tomography image (b).

**Figure 3:**
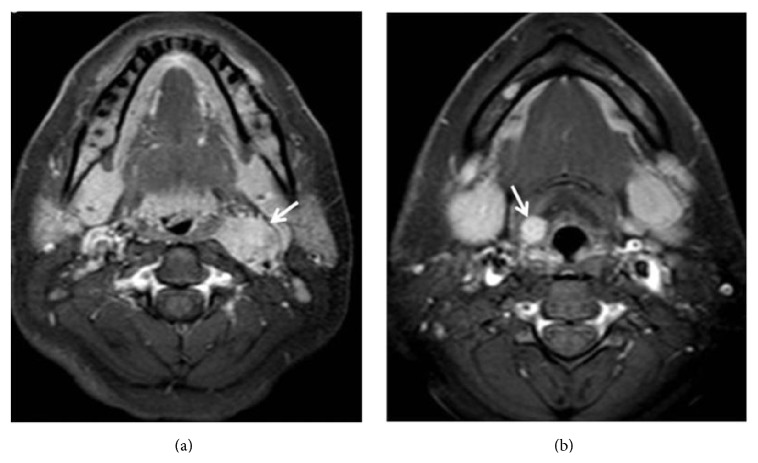
Enhanced lesions (arrows) are seen at left carotid bifurcation (a) and right supraglottic larynx (b) in axial contrast enhanced T1 weighted turbo spin echo spectral fat saturation inversion recovery image (T1 TSE SPIR).

On ultrasound (US) exam well defined, oval shaped, hypoechoic, solid, 25 × 32 mm hypervascular mass was seen at the left carotid bifurcation (Figures 1(a) and 1(b)). Splaying of the internal and external carotid arteries and hypervascularity suggested that paraganglioma and thereby fine needle aspiration biopsy were not applied. Contrast enhanced computed tomography (CT) revealed hypervascular mass in the same region (Figure 2(a)). Additionally, well defined, ovoid, 10 × 12 mm hypervascular mass was seen in the right preepiglottic space (Figure 2(b)). Magnetic resonance imaging (MRI) was performed for further characterization. MRG demonstrated that both lesions had similar imaging features including isointense on T1W, hyperintense on T2W images, and homogeneous intense enhancement (Figures 3(a) and 3(b)). Imaging findings were suggestive of multicentric HNP.

**Figure 4:**
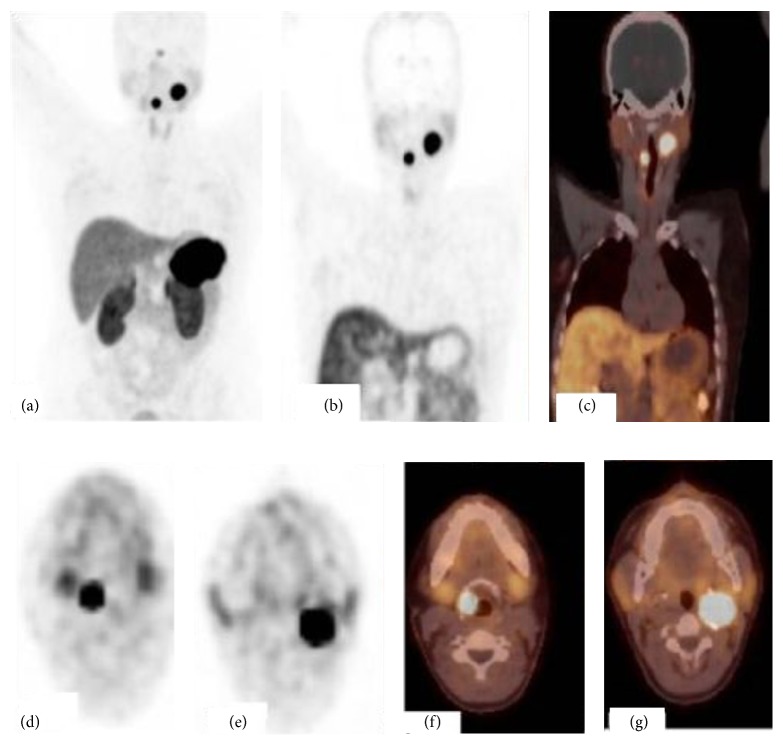
68Ga-DOTANOC PET/CT; maximum intensity projection (MIP) image (a), coronal PET and PET/CT fusion images (b, c), and axial PET and PET/CT fusion images (d, e, f, and g) show an intense uptake by the right laryngeal paraganglioma and left carotid body paraganglioma.

Because of multicentricity and positive family history, Gallium-68 (Ga-68) DOTA-peptide positron emission tomography/computer tomography (PET/CT) was performed to verify the diagnosis and disclose whether or not another focus of paraganglioma exists anywhere in the body. 68Ga-DOTANOC PET/CT showed the laryngeal lesion with intense tracer uptake (SUV max: 35.8) and carotid body paraganglioma (SUV max: 37.5) (Figures 4(a), 4(b), 4(c), 4(d), and 4(e)). 68Ga-DOTANOC PET/CT demonstrated no additional abnormality apart from these two lesions.

**Figure 5:**
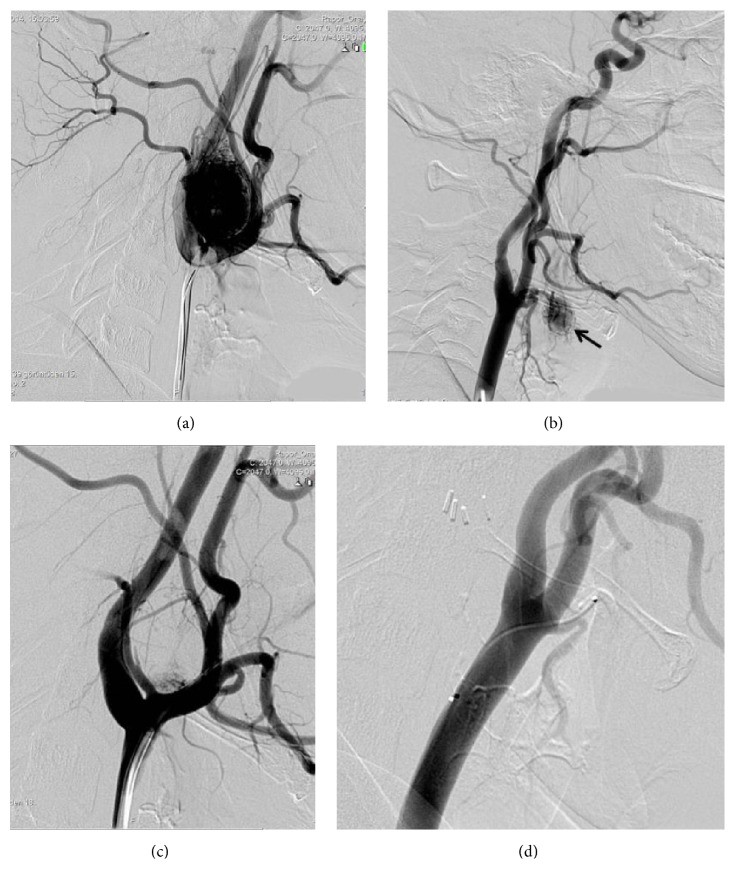
A lateral cervical angiogram from the left common carotid artery demonstrates the intense blush at the carotid bifurcation corresponding to the carotid body paraganglioma (a). Right common carotid artery lateral cervical angiogram shows significant tumor vascularity (arrow) at the larynx (b). Principal arterial feeder to the tumor from superior thyroid artery. Significant reduction is seen in tumor vascularity after embolization of the ascending pharyngeal and proximal occipital artery with polyvinyl alcohol particles measuring 255 to 350 (c). Superselective catheterization of the superior thyroid artery after embolization shows complete devascularization (d).

Digital subtraction angiography (DSA) demonstrated right superior thyroid artery supplied laryngeal mass and left ascending pharyngeal artery supplied mass at carotid bifurcation (Figures 5(a) and 5(b)). The mass located carotid bifurcation was embolized using microcatheter with 250–350 sized polyvinyl alcohol particles (Figure 5(c)). Balloon occlusion test of the left internal carotid artery was performed and it was tolerated without any neurological deficit. Surgery was performed following embolization.

The pathologic diagnosis was paraganglioma. Microscopically, the well encapsulated tumor made characteristic clear cell nests with Zellballen pattern. Immunohistochemical analysis showed tumor cells positive for chromogranin and S-100 (sustentacular cells). Staining for Ki67 showed <1% positive cells. Five months later, second operation was performed for laryngeal tumor following superior thyroideal artery embolization (Figure 5(d)). The diagnosis of laryngeal paraganglioma was confirmed on histopathology and immunohistochemistry.

## 3. Discussion

Multiple paragangliomas may occur in 40% of familial paragangliomas and 10% of sporadic tumors [2]. Chase described first familial paraganglioma in two sisters who both presented with bilateral carotid body tumors in 1933 [7]. Familial paragangliomas account for 10% of cases [8]. Multiple paragangliomas may arise synchronously or metachronously. Rubin et al. [5] reported that typically laryngeal paragangliomas are not found in patients with multicentric or familial paragangliomas. The case presented here is the first case of laryngeal with carotid body paraganglioma in a patient with positive family history.

Larynx is a very rare location for paragangliomas of the head and neck. The majority of laryngeal paragangliomas arise in the supraglottic larynx. They occur most commonly in the fourth to sixth decades and show a female preponderance (1 : 3), unlike other neuroendocrine tumors of the larynx [9]. Hoarseness, dysphonia, cough, and dyspnea are the commonest presenting symptoms. Its clinical features are similar to other laryngeal neoplasms. On laryngoscopy, tumors are usually seen as submucosal masses. The case presented here was asymptomatic and there were not any abnormalities on the laryngoscopic examination. There is only one asymptomatic case who presented with shortness of breath and airway compromise after intubation due to laparoscopic cholecystectomy [10].

Laryngeal paragangliomas must be differentiated from other neuroendocrine tumors of the larynx, including typical carcinoid, atypical carcinoid, small cell neuroendocrine carcinoma, and large cell neuroendocrine carcinoma [11]. Paraganglioma and other neuroendocrine tumors have different biological behavior and prognosis; thus treatment strategies are different. Because of their overlapping histological appearance, differential diagnosis is based on immunohistochemistry. Endoscopic biopsy is controversial due to the submucosal and vascular nature of the lesion. Deep biopsy may cause uncontrolled bleeding and require tracheotomy. Diagnostic imaging plays a critical role in the biopsy decision and clinical management of the patient with laryngeal submucosal mass. Differential diagnosis of laryngeal submucosal mass includes hemangioma, schwannoma, chondroma and chondrosarcoma, and minor salivary gland tumors such as adenoid cystic carcinoma [12–14].

Doppler US is a first-line imaging technique in patients with suspected carotid body paraganglioma. Well defined solid mass at the carotid bifurcation, splaying of the internal and external carotid arteries, and high vascularity are typical findings of these tumors. US cannot demonstrate jugulotympanic and most of vagal paragangliomas due to their farther cephalad localization. Contrast enhanced computed tomography is a useful technique for defining bone changes in patients with jugular paraganglioma such as irregular widening of the jugular foramen with cortical erosion and permeative bone destruction. MRI provides better tissue characterization and allows detailed evaluation of tumor localization, size, vascularity, its relationship with major arteries and surrounding structures, and presence of intracranial extension. The typical MRI finding of paragangliomas is “salt and pepper” appearance that results from slow flow or hemorrhage and signal void areas due to high velocity flow, respectively. CT and MRI may also show asymptomatic synchronous HNP especially in patients with familial paraganglioma. Imaging findings of laryngeal paraganglioma are similar to other HNP. In our case, similar imaging features of both lesions confirmed diagnosis of multiple paragangliomas.

DSA is most commonly used for preoperative vascular mapping and tumor embolization rather than diagnosis. Also, balloon occlusion test can be performed to assess collateral cerebral perfusion in patients with carotid body paragangliomas to determine the risk for ischemia and stroke following ICA ligation.

68Ga-DOTA-peptide PET/CT has higher sensitivity and advanced spatial resolution for the detection of well differentiated neuroendocrine tumors than conventional somatostatin receptor scintigraphic agents. Apart from characterizing a lesion as neuroendocrine tumor, 68Ga-DOTA-peptide PET/CT can reveal extension of disease and exclude synchronous and metastatic lesions. 68Ga-DOTA-peptide PET/CT study may further assist in choosing the right treatment and selection of patients for peptide receptor radionuclide therapy with 177 Lu or 90 Y labeled peptides in such patients with extensive disease.

In conclusion, radiologists should keep in mind that paragangliomas may arise in head and neck region as synchronous or metachronous multiple tumors. All areas within the images should be carefully evaluated to avoid missing asymptomatic lesions. Earlier detection and treatment of these tumors can reduce morbidity and mortality rates of surgery.
